# Epidemic “Bowel Complaint”

**Published:** 1850-02

**Authors:** George Little

**Affiliations:** Ann Arbor, Michigan


ART. III.—*Epidemic "Bowel Complaint."* By GEO. LITTLE, M. D., Ann Arbor, Michigan.

SIR: Having seen an article in your last number on epidemic "Bowel Complaint," I am induced to forward you the particulars of two cases lately under my care, which terminated fatally, under the impression that the more investigation is elicited of an obscure and unmanageable disease, the better for the interests of the community.

John Gilslenard, aet 34, of phlegmatic temperament, was attacked with diarrhœa of a sanguinolent character, restlessness, thirst, and pain in the umbilical region. Saw him the following morning, and put him on small doses of calomel, opium, and camphor, with compound chalk mixture after each stool; ordered stuping, and mustard cataplasm over seat of pain. Next day, finding the evacuations less frequent, but unchanged in character, I treated him with acetate of lead and opium by mouth and injection. At this stage, great irritability of stomach existed. Nothing could be retained, thirst great, pulse, 110, and feeble, tongue dry and chapped, dejections again occurring every ten minutes, of masses of lymph in bloody serum. The following day he was somewhat improved; dejections, though bloody, were more of a mucous character, and occurred at intervals of two hours. Stomach quieter, and pain removed. At this period, by advice of an older physician just called in, the treatment was laid aside, and three drops of laudanum, in a little toast water, given after each stool, with one grain of calomel, and one-fourth grain opium every sixth hour. Symptoms became worse, and though the former treatment was again resorted to, the patient sunk into collapse the fourth morning after his attack.

Michael Clarkins, at 51, of bilious temperament, mason by trade, was attacked with diarrhœa, which he thinks was brought on by cold. This he neglected for a week. I found him similarly affected as the former case; the same character of evacuation, flocculi of lymph in a large quantity of bloody serum without feculent odor, pain over umbilical region, though not increased by pressure, fever and thirst much less. Treated him at first with blue pill and Dover's powder, following it up with a draught of castor oil and tinct. rhubarb, which gave transient relief.

Next day, finding the symptoms not much relieved, put him on small doses of calomel, acetate of lead and opium, with compound chalk mixture and catechu after each stool, stupes, cataplasm, &c. to seat of pain. Irritability of stomach set in the second day; controlled this with the mist. camphoræ and mag., and allowed him small quantities of beef tea. Found him the following day rather better, evacuations assuming a feculent character, stomach quieter, pain entirely relieved, frequent evacuations from stomach. By the advice of friends, another physician was called in, who recommended a change of the treatment for a slightly purgative one of rhubarb and magnesia. This I acceded to, but found, after nearly two day's trial, matters were worse; stools took on their former character, and every thing was rejected from the stomach, medicine and food. A third physician was added in consultation, and it was determined, conjointly, that decided doses of calomel should be given, with a view to its sedative, purgative, and afterward, alterative effects. Accordingly, ten grain doses were given at an interval of an hour, till three were taken. Afterwards five grains every third hour, till six were taken. The first three doses were retained, the last rejected. Stools next day were bilious, with a good deal of dark, feculent matter, mucus, and blood. He appeared somewhat relieved after this, but all his bad symptoms set in towards evening. Next morning emesis set in again with constant evacuations, dejections, sanguinolent as before; pulse more feeble, countenance anxious and dejected. Under this state of things it was suggested and resolved to desist entirely from medical, and rely on dietetic treatment. From this period, the stomach became altogether settled. Stools less frequent, though still the same, and it almost seemed as if nature was endeavoring to make a last rally. This patient, exhibiting at times the phases of recovery, and again relapse, ultimately sunk four weeks after his first attack, being seven days without advice, eleven days under medical treatment, and eleven under dietetic management.

That this disease puts on an intermittent character, the reflexion being upon the mucous membrane of the stomach and bowels, producing a state of congestion of its vessels and abnormal secretions, is an idea that may be fairly entertained, whether produced by malaria or not; and that a tonic febrifuge medicine, such as quinine, or iron, would be attended with happy results, I have no doubt, as I have witnessed good effects in protracted cases from such remedies. What, however, is to be done in the mean time, with a hemorrhage that saps the foundation of life, exhausts her resources by the abstraction of lymph, blood and serum, and numbers the hours of the patient, if unchecked! Are we to indulge in theories on this and that mode of treatment, while the patient is evidently sinking before us, or, rather, are we to resort to those remedies known to be efficacious in hemorrhage, and put a stop, at least, to this, and make a sudden impression upon the disease, and then use our tonic remedies? I have witnessed such good results from the calomel, acetate of lead, and opium treatment, if steadily persevered in, with occasionally starch and laudanum injections, that I have great reliance on it. Quinine and iron are good auxiliaries, when the urgent symptoms subside.

Should these hastily written, and ill-digested remarks shed, through a spirit of inquiry, more light to brethren on an obscurely understood and dangerous disease, I shall feel that I have not written in vain.

Yours, &c. GEO. LITTLE, M. D.

